# S494 O-glycosylation site on the SARS-CoV-2 RBD affects the virus affinity to ACE2 and its infectivity; a molecular dynamics study

**DOI:** 10.1038/s41598-021-94602-w

**Published:** 2021-07-26

**Authors:** Shadi Rahnama, Maryam Azimzadeh Irani, Mehriar Amininasab, Mohammad Reza Ejtehadi

**Affiliations:** 1 Institute for Nanoscience and Nanotechnology, Sharif University of Technology, Tehran, 14588 Iran; 2 Faculty of Life Sciences and Biotechnology, Shahid Beheshti University, Tehran, Iran; 3 Department of Cell and Molecular Biology, School of Biology, College of Science, University of Tehran, Tehran, Iran; 4 Department of Physics, Sharif University of Technology, Tehran, 14588 Iran

**Keywords:** Biotechnology, Computational biology and bioinformatics, Drug discovery, Molecular biology

## Abstract

SARS-CoV-2 is a strain of Coronavirus family that caused the ongoing pandemic of COVID-19. Several studies showed that the glycosylation of virus spike (S) protein and the Angiotensin-Converting Enzyme 2 (ACE2) receptor on the host cell is critical for the virus infectivity. Molecular Dynamics (MD) simulations were used to explore the role of a novel mutated O-glycosylation site (D494S) on the Receptor Binding Domain (RBD) of S protein. This site was suggested as a key mediator of virus-host interaction. By exploring the dynamics of three O-glycosylated models and the control systems of unglcosylated S4944 and S494D complexes, it was shown that the decoration of S494 with elongated O-glycans results in stabilized interactions on the direct RBD-ACE2. Calculation of the distances between RBD and two major H1, H2 helices of ACE2 and the interacting pairs of amino acids in the interface showed that the elongated O-glycan maintains these interactions by forming several polar contacts with the neighbouring residues while it would not interfere in the direct binding interface. Relative binding free energy of RBD-ACE2 is also more favorable in the O-glycosylated models with longer glycans. The increase of RBD binding affinity to ACE2 depends on the size of attached O-glycan. By increasing the size of O-glycan, the RBD-ACE2 binding affinity will increase. Hence, this crucial factor must be taken into account for any further inhibitory approaches towards RBD-ACE2 interaction.

## Introduction

Severe Acute Respiratory Syndrome Corona Virus 2 (SARS-CoV-2) is a positive-sense single-stranded RNA virus which caused the pandemic of COVID-19 that is still going on. As of 31st March 2021, 128 million cases and 2.8 million deaths of COVID-19 were reported worldwide. In Wuhan, China, the virus is believed to transmit from bat to human^1^ and underwent several inter and intra species passages. This large-scaled pandemic motivated several scientific groups to control the virus’s spread and treat the infected patients by all means. The researchers are mainly focused on the phylogeny and origin of SARS-CoV-2^1^, Structural assembly and the dynamics of virion-host cell proteins^2–6^ and the experimental efforts to obtain a SARS-CoV-2 neutralizing antibody^7,8^.

**Figure 1:**
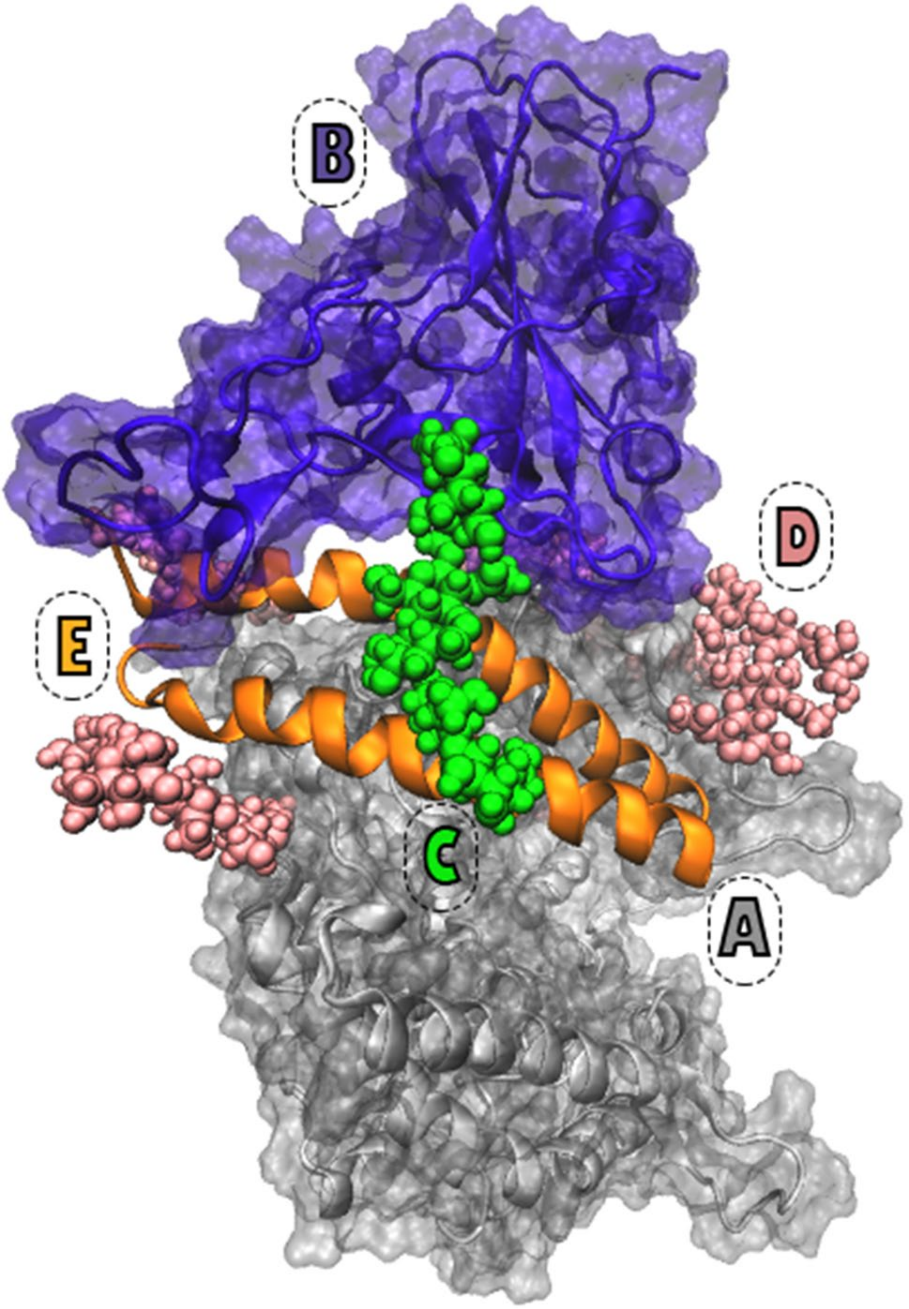
The extracellular domain of human ACE2 and RBD of SARS-CoV-2 S protein is shown with gray and purple, respectively (**A**,**B**). The RBD attached O-glycan is shown with green spheres (**C**). For the sake of simplicity, only the N-glycans in the proximity of RBD-ACE2 interface are shown with pink spheres. Visualization of the complex with full presentation of the N-glycans can be found in Fig. S1 (**D**). Two O-glycan interaction sites H1 and H2, are shown with orange (**E**).

The SARS-CoV-2 virion assembly and its infusion into the human cell are similar to the one known for SARS-CoV^2^. The fusion protein known as the spike (S) protein interacts with ACE2 on the host cell via its Receptor Binding Domain (RBD)^3,9^ (Fig. 1).The RBD-ACE2 interaction takes place via four $\beta$ strands on RBD (*β*4–*β*7) and two $\alpha$ helices on ACE2 binding interface (H1,H2)^3^ (Fig. 1). Electron microscopy and X-ray crystal structures elucidated the active form of S protein which assembles as a trimer^2^, and one RBD of the S protein in complex with ACE2^3^. The structures of S protein in the apo and ACE2 bound complexes show that RBD adopts a 3D fold similar to the RBD of SARS-CoV^2^. However, six novel mutations on SARS-CoV-2 RBD could lead to different binding affinity to ACE2^1^. Speculations regarding SARS-CoV-2-ACE2 binding affinity suggested two different scenarios. One reported a dramatic increase (up to 20-fold) in the binding affinity of SARS-CoV-2 to ACE2 compared to SARS-CoV^2^. While, the other suggests a similar binding affinity for SARS-CoV-2 and SARS-CoV to ACE2^3,10^. Either way, the mutations in SARS-CoV-2 RBD certainly results in attenuated binding affinity of all designed inhibitors which successfully work on SARS-CoV^2^. This was a challenging issue for anti-SARS-CoV-2 drug design attempts since the pandemic has started.

In addition to the 3D structures of RBD-ACE2 complex, several recent Molecular Dynamics (MD) studies addressed the complex dynamics to design peptide inhibitors. That block the ACE2-RBD interaction and evaluate the currently targeted binding epitopes’ accessibility for further improvements^4,11,12^. Earlier MD studies explored the effect of pH^13^ and temperature^14^ on the dynamics of SARS-CoV-ACE2 complex.To the best of our knowledge, there is currently no approved therapeutic inhibitor for SARS-CoV-2.

A pioneering study showed that the observed mutations at the junction of S1 and S2 subdomains of the SARS-CoV-2 S protein result in the emergence of a polybasic cleavage site adjacent to three O-glycosylation sites which are novel to SARS-CoV-2 and were not observed in any other related virion^1^. It was proposed that O-glycosylation (addition of glycan building blocks to hydroxyl oxygen of Ser/Thr residues which is an enzymatic post transnational modification^15^) could lead to recognition of polybasic cleavage site by Furin enzyme and thus resulted in higher infectivity and broadens the host range of the virus^1^.

A Recent comprehensive experimental mass-spectrometry study on both N- and O-glycans attached to the spike protein did not report O-glycosylation of the S494. However, two O-glycosylated sites (S325/T323) were reported on the RBD^16^. Another recent computational study that was validated by experimental data reported that the S325/T323 O-glycosylation sites that were observed by Shajahan et al. is present with 11% frequency along side other O-glycosylation site that were decorated with lower frequencies^17^.

Six mutations have been reported on the RBD of SARS-CoV-2 compared to the SARS-CoV. One of these six occurring mutations is D494S on the RBD of S protein. Based on the proposed mechanism for the O-glycosylation of Furin cleavage site of SARS-CoV-2^1^, and the experimental strucutral data that was mentioned above^16,17^, decoration of Serine494 of RBD with the O-glycan is also plausible even though it is not experimentally confirmed.

Our comprehensive review of literature on pathology and glycosylation pattern of the envelope proteins of all other coronaviruses and their close relatives showed strong evidence for the critical role of O-glycosylation in regulation and immune evasion of the viruses^1,18–20^. Also, former studies showed that the O-glycosylation of the membrane (M) protein of Mouse Hepatitis Virus (MHV) triggers a lower interferon level than the N-glycosylated M protein^18^. 3A protein of the MHV, an accessory protein to the M protein is also known to be O-glycosylated. Thus, it could be shielded from Peptide-N Glycosidase F (PNGase F) which cleaves most monosaccharide attached to protein. On the other hand, a complex nontemplate mechanism of the O- glycosylation could occur via several Polypeptide N-acetyl Galactoseamine Transferases (PPGalNAcTs) specific to various tissues was shown to be a common mechanism for the pathology of several high-evasion viruses such as Influenza^21^. In fact, the experimental findings suggest that the mucin proteins of human respiratory airways are heavily O-glycosylated with carbohydrate chains^22–24^. These dense O-glycan chains are known to trap invading microorganisms such as various strains of bacteria^25^.

Hence the lack of O-glycosylation in the currently available structures of RBD-ACE2 complex could be related to the expression host of the proteins and various glycosylation patterns in different cell lines.

Putting all the information mentioned above together, it is highly plausible that D494S mutation has an evolutionary origin and leads to O-glycosylation of RBD in the SARS-CoV-2. Hence, the O-glycosylation could be the overlooked factor for the SARS-CoV-2 increased binding affinity to ACE2, explaining the virus’s high infectivity in the human host and exposing people with a higher level of blood sugar at higher risk^26^. Our observations may explain the reason for the higher vulnerability of diabetic patients to infection.

**Figure 2:**
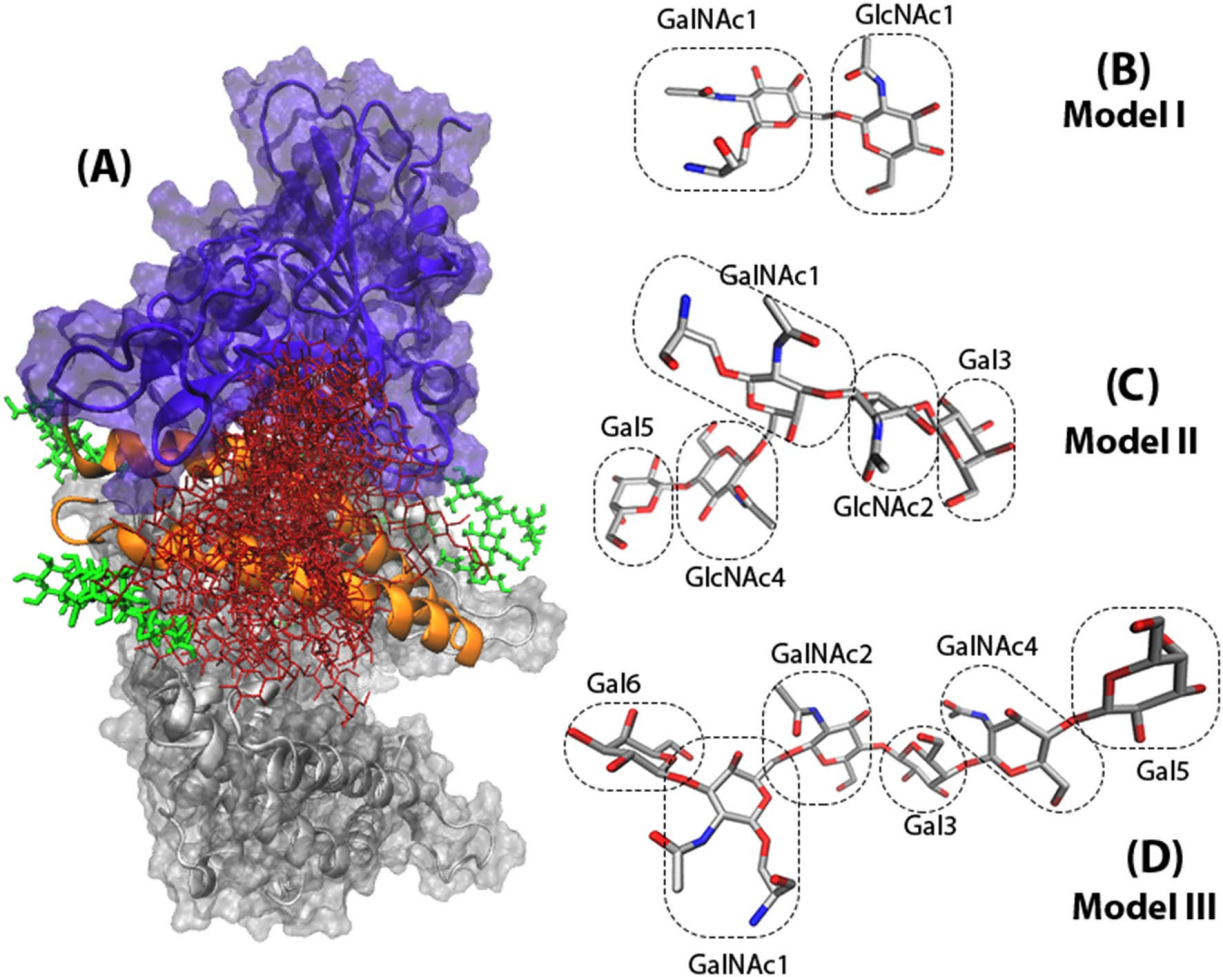
(**A**) The overlaid presentation of O-glycosylated RBD-ACE2 complex is shown with red sticks. The extracellular domain of human ACE2 and RBD of SARS-CoV-2 S protein is shown with gray and purple, respectively. For the sake of simplicity, only the N-glycans in the proximity of RBD-ACE2 interface are shown with green sticks. Visualization of the complex with full presentation of the N-glycans can be found in Fig. S1. O-glycan models I (**B**), II (**C**) and III (**D**) are shown with atom name sticks.

Herein, we explored this possibility by modeling the O-glycosylated RBD structure and its dynamics in interaction with ACE2 (Fig. 2). We showed that O-glycosylation does indeed increase SARS-CoV-2 RBD-ACE2 binding affinity. And attachment of the elongated O-glycans will increase the binding affinity. Furthermore, we tested the validity of these observations by considering the structural role of S494 substitution with D in the SARS-CoV-2 RBD to entirely eliminate the structural role of S494 in the dynamics.

In summary, this work provides strong evidence that O-glycosylation of SARS-CoV-2 RBD in the respiratory airways leads to stronger interactions between the virion and the host cell receptor.

Hence, further inhibitor design attempts must take the detailed atomistic glycan-protein interaction reported here into account as a critical factor for future investigations.

## Methods

### Model building of RBD-ACE2 complexes

The crystal structure of SARS-CoV-2 RBD (residue 333 to 526) in complex with the extracellular domain of ACE2 (residue 19 to 615) (PDB ID: 6M0J) was used as the starting structure. Zn^2+^ and Cl^−^ ions which were resolved in the crystal structure for their crucial role in stabilizing ACE2 structure were also preserved at S1 and S2 subunits of ACE2. All Aspargines located within N-glycosylation motifs from RBD (Asn343) and ACE2 (Asn53, Asn90, Asn103, Asn322, Asn432, and Asn546) were glycosylated. The GLYCAM online builder^27^ was used to attach the oligosaccharide N-glycans to each site. This oligosaccharide model was selected as a primary model of glycosylation which occurs in normal human cells^15^. Supporting Table S1 provides details on the structure of attached N-glycans.

O-glycosylation of Serine494 of RBD was carried out by attaching three models of core and branched typical human O-glycans using GLYCAM online builder^27^. Models I, II, and III are consist of two, five, and six monosaccharide units, respectively (Fig. 2).Details of all the glycosidic linkages and the bonding atoms can be found in Fig. S.2 and Table S.2. These O-glycan models were attached to RBD, while RBD-ACE2 complex was kept fully N-glycosylated (Fig. 1). Control system of RBD-ACE2 model without the attached O-glycan was also considered and will be referred to as model S. The model with the Serine 494 substitution with Aspartic acid in the RBD was prepared to completely eliminate the structural role of D494S mutation of SARS-CoV-2 in the RBD-ACE2 interactions.This model will be referred to as model D.

### Molecular dynamics simulations

All systems were solvated using TIP3P water model^28^ and were neutralized by attaining a buffered environment in 150 mM NaCl. The resulting water box approximately has a dimension of $121 \times 111 \times 149$ Å$^3$ and are typically consist of 190000 atoms each. All systems were parameterized with the CHARMM36m force field^29,30^, and NAMD2.12^31^ was used to perform the MD simulations.All of the production runs were carried out under periodic boundary conditions under NPT ensemble, while employing Langevin thermostat and Nose-Hoover Langevin piston method to keep systems’ temperature and pressure at 310 K and 1 bar, respectively. A cutoff of 12 Å was assigned for computation of short range nonbonded van der Waals interactions. The particle mesh Ewald method^32^ was used for long-range electrostatic interactions. R-RESPA multiple time-step schemes were used for the integration of motion equations^31^. Using this integrator, the Lennard-Jones interactions and bonded ones were updated every step and electrostatic interactions every two steps. Along with the restraining of all covalent hydrogen bonds by SHAKE, the time steps for integration were set to 2fs. Final models of all complexes were minimized for 5000 steps to remove any steric clashes. The systems were then equilibrated for 0.5 ns under the NVT ensemble followed by another 0.5 ns relaxation under the NPT ensemble. The position of atoms in the complex (proteins, Zn^+2^ and Cl^−1^ ions) was restrained using a harmonic potential with spring constant k = 1 kcal/(mol Å$^2$). Production runs were then performed in three replicates of 100 ns for models S, I, II and III. For the control model D, two simulations of 100 ns was carried out. (with the exception one simulation for the control model D). By selecting the PDB ID 6M0J crystal structure of the complex, performing several minimization steps and extensive equilibration, the structure of the ACE2-bound RBD is very well relaxed. Thus, the Orientation of the O-glycosylated RBD-ACE2 is reliable.

During the production runs, all restraints on the protein were turned off, while dummy bonds between Zn^+2^ and Cl^−1^ ions and their adjunct atoms within radius of 3 Å were kept intact.

### Binding free energy calculations

The binding free energy between RBD and ACE2 was calculated with Molecular Mechanics Poison Boltzmann Surface Area (MMPBSA) method. Here we used CaFE pipeline tool^33^ on 250 frames from the last 10 ns of MD simulations and an ensemble-averaged over all replicates of each model. For electrostatic energy calculations, APBS method^34^ was used. The dielectric constants for solvent and protein were set to 80.0 and 1.2 respectively (More details could be found in Supplementary method).Probe radius for calculation of SASA was set to 1.4 Å.

### MD simulations analyses

Root Mean Squared Deviation (RMSDs), Root Mean Squared Fluctuation (RMSFs), Solvent Accessible Surface Area (SASA) and the distances between the interacting pairs of residues, RBD and ACE2, H1 and H2 were calculated by tcl scripts taking advantage of VMD^35^. RMSD of each system was calculated by considering the backbone atoms (C$_{\alpha }$, C, and N). RMSF and the distance among the geometric centers of domains were calculated for C$_{\alpha }$ atoms. SASA was calculated bey selecting the interface residues between RBD (405-505) and ACE2 (19-99). All trajectories were fitted to the starting conformation. All the plots were generated by Matplotlib^36^ library of Python^37^ and the figures by VMD^35^.

## Results and discussion

### Dynamics of the O- and N- glycosylated RBD-ACE2

It is worth mentioning again that as all the systems are fully N-glycosylated here, the N-glycans’ global effect is explicit in the dynamics. However, the interpretation of results is mainly focused on O-glycosylation. All systems are simulated in three replicates except control model D with two replicates. In each RMSD plot, the probability distribution function (PDF) shows the the fluctuations of RMSD data which is interpreted as flexibility in this text. One should note that the RMSD plots are not presenting the stability of the complex in terms of the evolutionary dynamics and they only represent the fluctuations of RMSD within the limited time-scale of the simulations.

**Figure 3:**
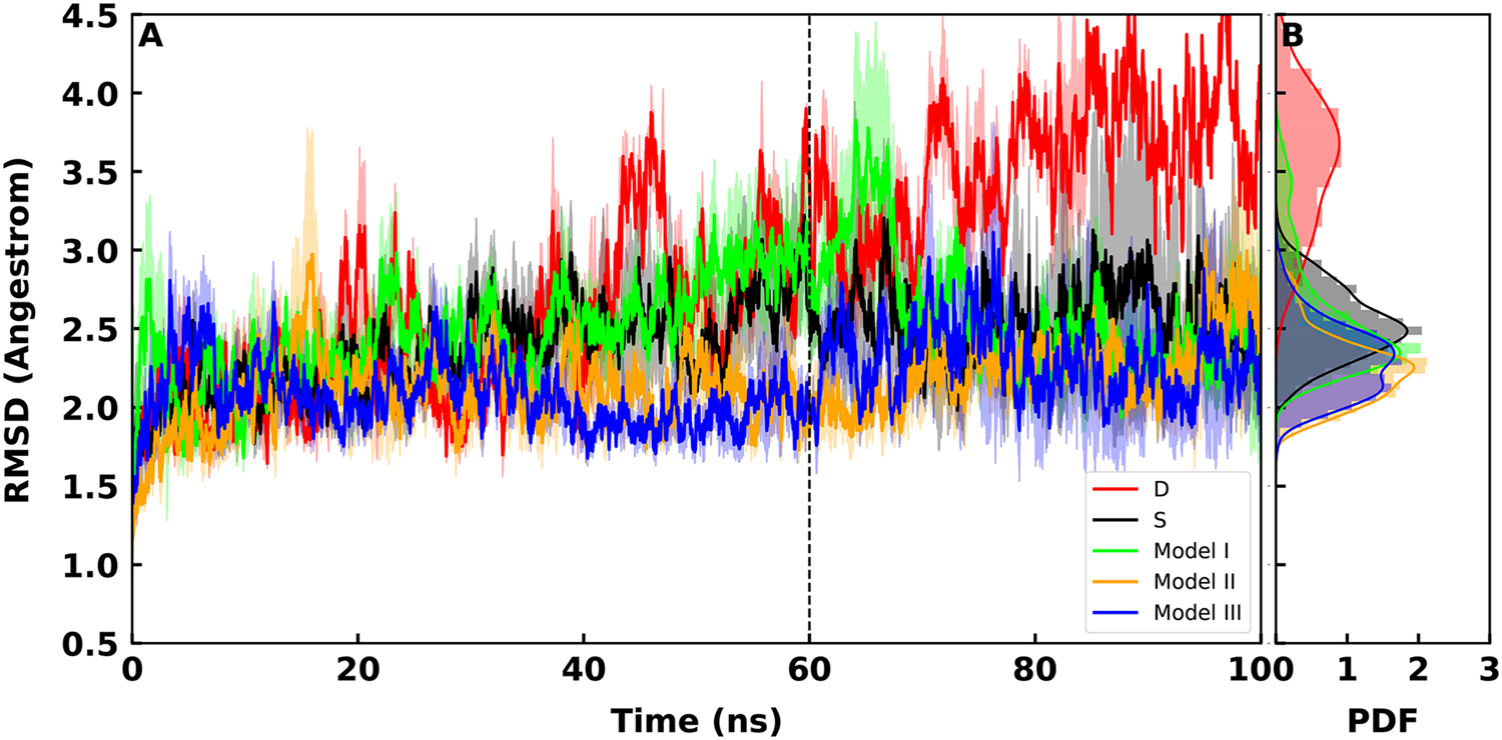
(**A**) Average backbone RMSD plots for RBD-ACE2, calculated from all replicates of each system and are shown for model S (black), model I (lime), model II (orange) and model III (blue). Light shades around each plot presents standard error for each calculation. RMSD of model D is shown in red. (**B**) Probability Density Function (PDF) of RMSD sampled over the last 40 ns (dashed line) of the simulations are shown in histograms.

RMSD plots of RBD-ACE2 complex simulations show that among three O-glycosylated systems, models III ($\sigma = 0.049$), II ($\sigma = 0.051$) and S ($\sigma = 0.048$) show similar flexibility while model I ($\sigma = 0.138$) comes just after. (Fig. 3 A,B). Model D ($\sigma = 0.203$) shows dramatically high fluctuations in the RMSD values (Fig. 3). This observation suggest that the D494S mutation stabilizes the RBD-ACE2 interaction in SARS-COV-2 regardless of the O-glycosylation. The histograms of the PDF for RMSD values show a clear decrease in the peaks values of models III, II compared to models I, S and D (Fig. 3).

This observation is in agreement with several other experimental and comuputational studies that reported the increased stability of the glubular and transmembrane protein complexes upon glycosylation with elongated glycans^38–40^.

**Figure 4:**
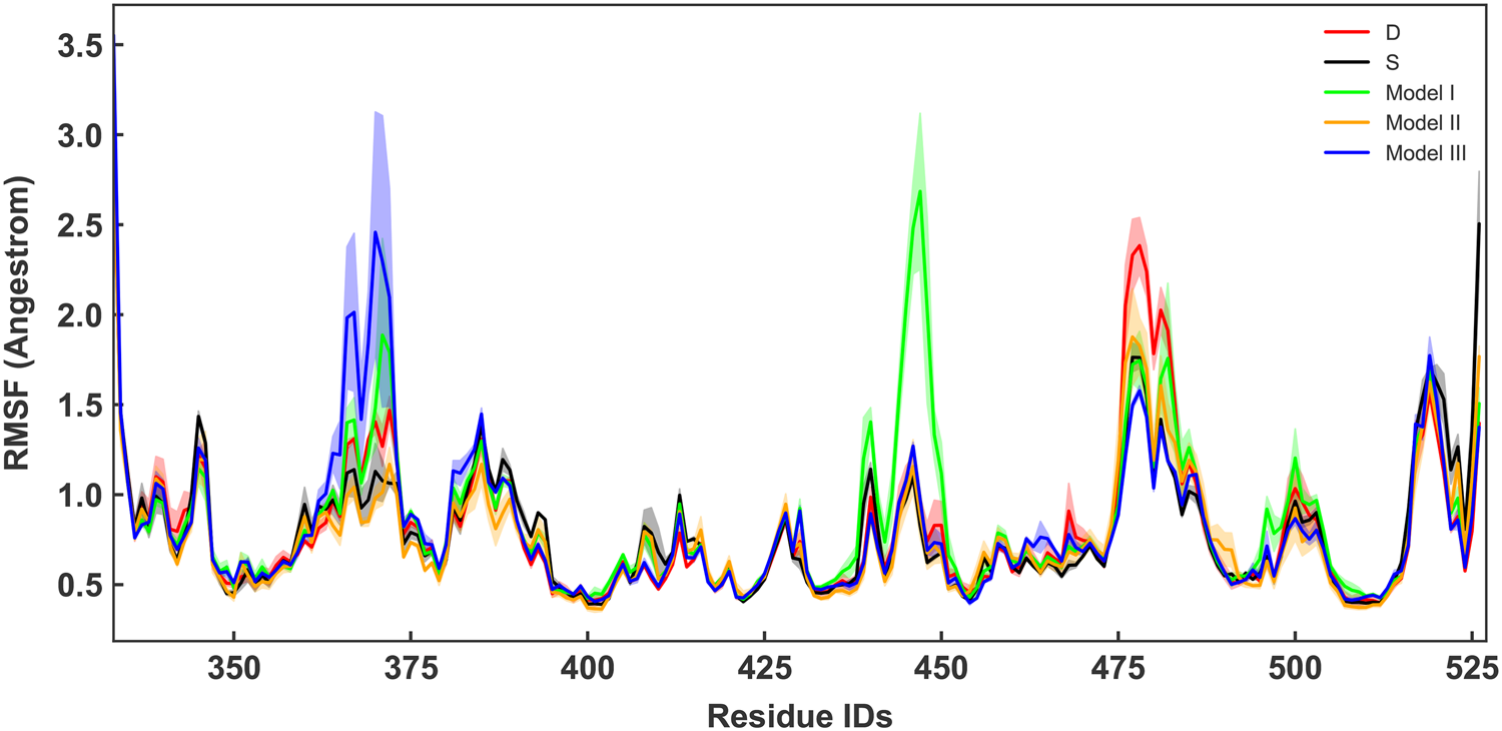
RMSF per residue of RBD calculated from all replicates of each system and are shown for model S (black), model I (lime), model II (orange) and model III (blue). Light shades around each plot presents standard error for each calculation. RMSF of model D is shown in red.

**Figure 5:**
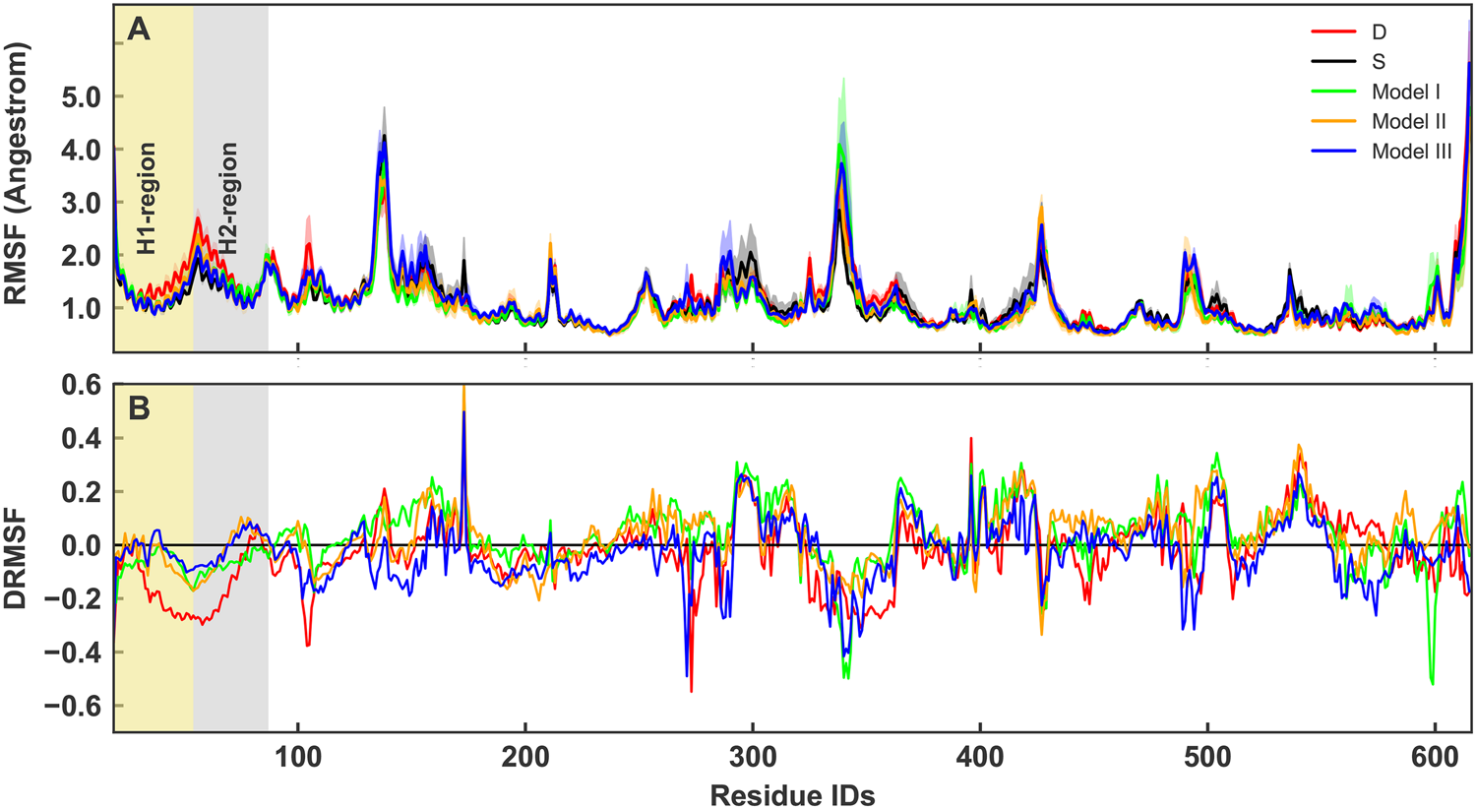
(**A**) RMSF per residue of ACE2 calculated from all replicates of each system and are shown for model S (black), model I (lime), model II (orange) and model III (blue). Light shades around each plot presents standard error for each calculation. RMSF of model D is shown in red. The transparent boxes indicates H1 and H2. (**B**) The relative difference in RMSF, $D_{\rm{RMSF}}$ is shown per residue for all the models (with the same color codes).

Overall RMSF values of the RBD show that different models present high peaks in RMSF at different locations (Figs. 4, 5). However, calculation of the average RMSF for the direct interface residues^41^ showed the lowest values for models III an II, while model S comes after (Table 1). Model D shows the hightest RMSF values (Table 1). This is supportive of the RMSD results that showed model D as the most flexible complex. The most dramatic increase of fluctuations occurs in model I residues 430 to 450 of RBD (Fig. 4). Two residues (GLY446 and Tyr449) within this region are known to interact with the ACE2 directly^42^. Attachment of a two-units O-glycan to model I, destabilizes these interactions as the glycan is not long enough to form contacts with the neighbouring residues. These results are interesting since the experimental findings has shown that the O-glycans of the respiratory airways are often elongated oligosaccharides^25^.

**Table 1:** Average RMSF of interafce residues.

System	Model D	Model S	Model I	Model II	Model III
ACE2	1.299	1.247	1.332	1.216	1.216
RBD	0.962	0.843	0.980	0.906	0.832
RBD-ACE2	14.298	13.719	14.655	13.381	13.378

### Detailed interactions

**Figure 6:**
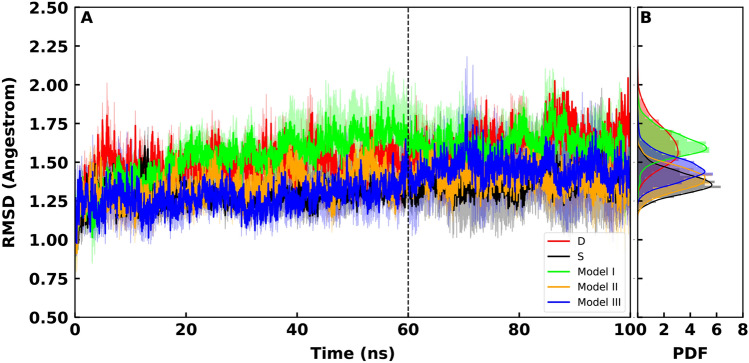
(**A**) Average backbone RMSD plots for Receptor binding RBD calculated from all replicates of each system and are shown for model model S (black), model I (lime), model II (orange) and model III (blue). Light shades around each plot presents standard error for each calculation. RMSD of model D is shown in red. (**B**) Probability Density Function (PDF) of RMSD sampled over the last 40 ns (dashed line) of the simulations are shown in histograms.

**Figure 7:**
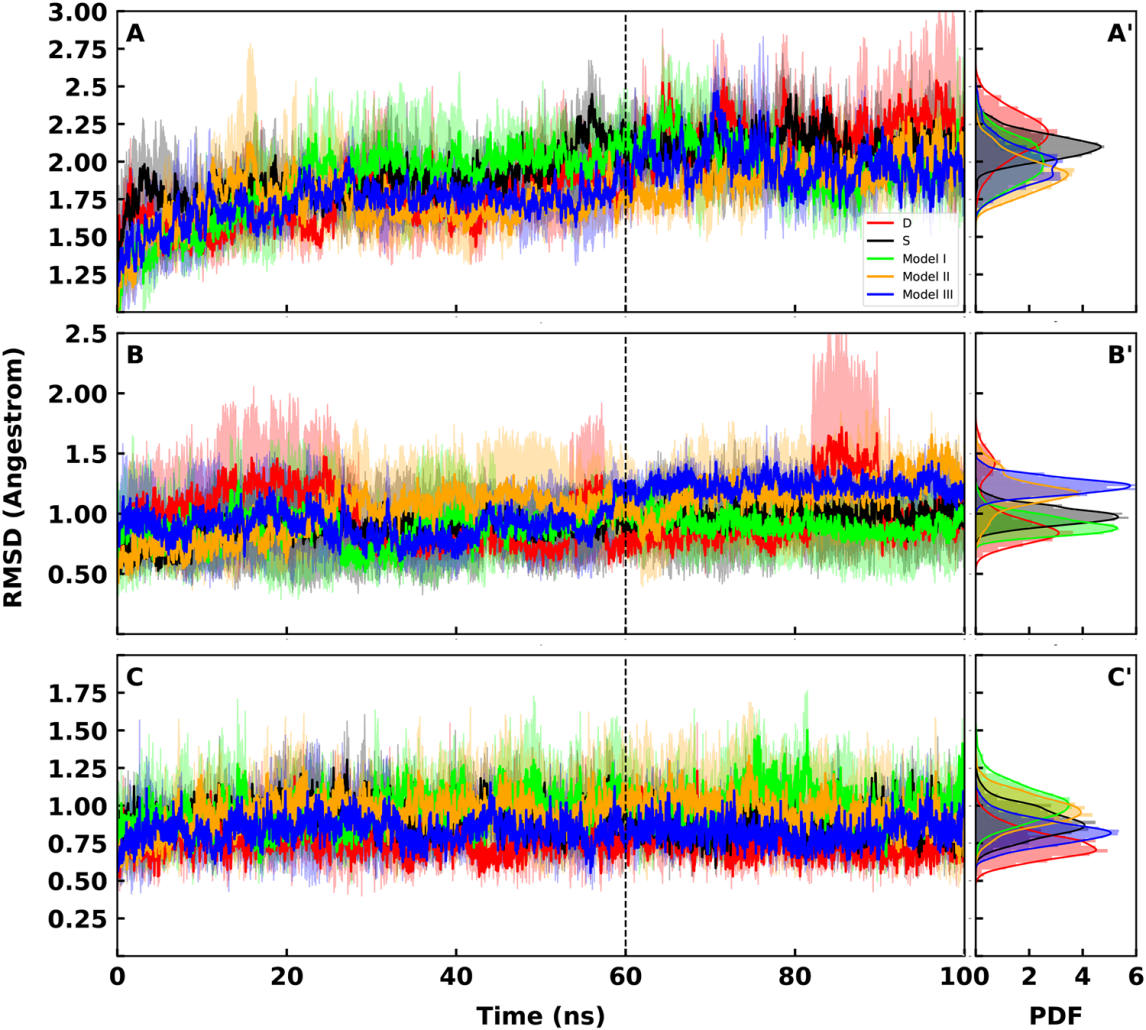
(**A**) Average backbone RMSD plots for ACE2 calculated from all replicates of each system and are shown for model S (black), model I (lime), model II (orange) and model III (blue). Light shades around each plot presents standard error for each calculation. RMSD of model D is shown in red. (**A**’) Probability Density Function (PDF) of RMSD sampled over the last 40 ns (dashed line) of the simulations are shown in histograms. (**B**) Average backbone RMSD plots for $\alpha$-Helix1 calculated from all replicates of each system and are shown for model S (black), model I (lime), model II (orange) and model III (blue). Light shades around each plot presents standard error for each calculation. RMSD of model D is shown in red. (**B**’) Probability Density Function (PDF) of RMSD sampled over the last 40ns (dashed line) of the simulations are shown in histograms. (**C**) Average backbone RMSD plots for $\alpha$-Helix2 calculated from all replicates of each system and are shown for model S (black), model I (lime), model II (orange) and model III (blue). Light shades around each plot presents standard error for each calculation. RMSD of model D is shown in red. (**C**’) Probability Density Function (PDF) of RMSD sampled over the last 40 ns (dashed line) of the simulations are shown in histograms.

Detailed investigation of the complex subunits showed that the average RMSD plots of RBD present similar flexibility in all systems ( $\sigma _I = 0.007$, $\sigma _{II} = 0.005$, $\sigma _{III} = 0.006$ and $\sigma _S = 0.005$) except model D that is the most flexible ($\sigma = 0.015$)(Fig. 6). But the effect of the attached O-glycans is be visible in ACE2 RMSD plots as the attached O-glycans interact with ACE2 $\alpha$-Helices. leading to a less flexible dynamics at the ACE2 interface (Fig. 7A–C). This observation also shows that the D494S mutation plays a critical role in stabilizing the RBD-ACE2 interaction.

ACE2 extracellular domain is a sizable receptor with about 600 amino acids; hence, it is expected that the overall RMSD of the receptor would not change much by the O-glycosylation of the RBD (Fig. 7A). As we aimed to study the alterations in ACE2-RBD interface, we calculated the RMSD of two $\alpha$-Helices (Figs. 7B,C) which are known as RBD interacting sites within ACE2 ^3^.

Average RMSD plots of H1 and H2 show that the O-glycosylated systems show different flexibility behaviour at these regions. Model I and III is less flexible and model II is more flexible in this region.($(\sigma _{III-H2} = 0.006 )>(\sigma _{III-H1} = 0.004)$ and $(\sigma _{I-H2} =0.013 )>(\sigma _{I-H1} =0.006)$ while $(\sigma _{II-H2} =0.01 )<(\sigma _{II-H1} = 0.02)$). Model III with the longest glycan is the least flexible (Fig. 7B). The two sharp peaks in RMSD plot of H1 for model D is resulted from high fluctuations of the dangling residues 19-22 at the N termini segment of H1 (Fig. S3) that is quite distant form the interface. On the other hand, H2 is more flexible in models I ($\sigma _{I-H2} =0.013$) and II ($\sigma _{II-H2} =0.01$) while model III ($\sigma _{III-H2} =0.006$) is more persistent (Fig. 7C) and presents the smallest flexibility values among other models. Although model S shows a similar dynamical pattern and $\sigma$ value with the O-glycosylated models in H1, here it shows a clear decreased in flexibility $(\sigma _{S-H2} =0.01 )$ compare to model III ($\sigma _{III-H2} =0.006)$). Model D shows the similar trend of dramatic fluctuations that we observed in the overall complex (Fig. 3) and RBD (Fig. 6) RMSD plots ($(\sigma _{D-ACE2} =0.021 ), (\sigma _{D-H1} =0.061), (\sigma _{D-H2} =0.009)$).

ACE2 RMSF plots support these observations, showing decreased fluctuations of H2 in the O-glycosylated simulations in comparison to model S (Fig. 5A). To highlight the differences, we have shown the relative differences compared to model S in Fig. 5B. The relative difference is presented as $D_{\rm{RMSF}}= \frac{RMSF_{0}-RMSF_i}{RMSF_{0}+RMSF_i}$ where $RMSF_i$ is per residue RMSF of each model. According to the definition more positive $D_{\rm{RMSF}}$ means lower relative RMSF.

**Figure 8:**
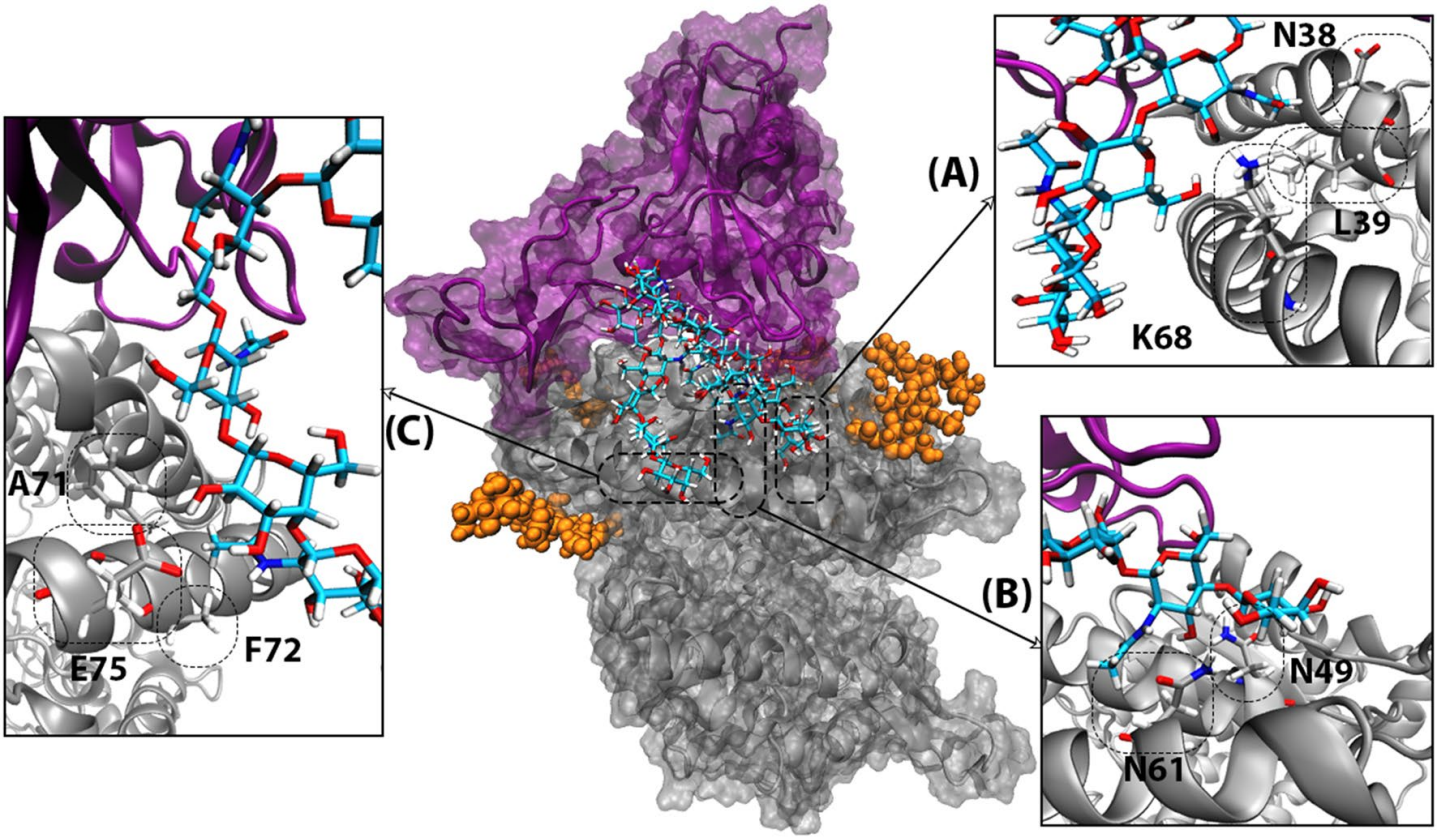
The extracellular domain of human ACE2 and RBD of the SARS-CoV-2 S protein are shown with gray and purple respectively. The RBD attached O-glycan is shown with atom name sticks. N-glycans in the proximity of RBD-ACE2 interface are shown with orange spheres. Persistent polar interactions between H1 and H2 of ACE2 and the attached O-glycan are shown in A-C.

Visualization of the dynamics shows that the O-glycan forms the most numerous polar contacts with H2 (five sites in H2 (N61,K68,A71,F72,E75) versus three sites in H1 (N38, L39, N49) Fig. 8.) In contrast, according to the crystal structures, H1 forms more contacts with RBD than H2 ^3^. RMSD plots of the attached O-glycans also show that the elongated models II and III are more flexible Fig. S2. That is due to the several polar interactions that they form with H2. One should note that the RBD is not O-glycosylated in the X-ray crystal structures. In fact that we see more interactions between O-glycan and H2 in our simulations suggests that the O-glycan keeps the RBD-ACE2 together by not interfering in the main ligand-receptor interactions that take place at H1.

### Biding affinity of O-glycosylated RBD to ACE2

**Figure 9:**
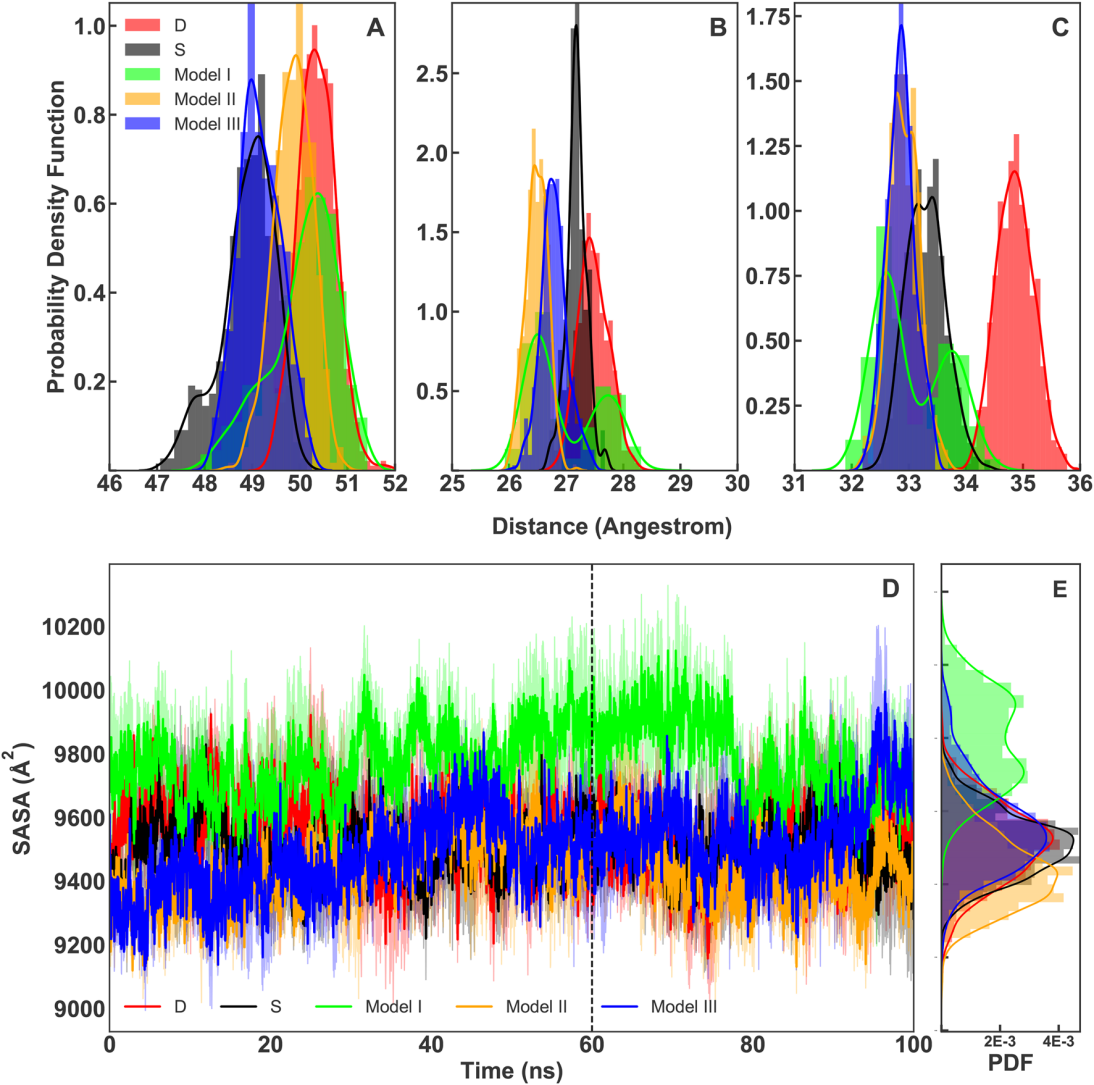
(**A**) PDF of RBD to ACE2 averaged distance distribution with a histogram and maximum likelihood gaussian distribution fit: calculated from all replicate of each system and are represented in black, lime, orange and blue for models S and I–III respectively. PDF of model D is shown in red. (**B**) PDF of RBD to H1 averaged distance distribution with a histogram and maximum likelihood gaussian distribution fit: calculated from all simulations of each system and represented in black, lime, orange and blue for models S and I–III respectively. PDF of model D is shown in red. (**C**) PDF of RBD to H2 averaged distance distribution with a histogram and maximum likelihood gaussian distribution fit: calculated from all simulations of each system and represented in black, lime, orange and blue for models S and I–III respectively. PDF of model D is shown in red. Sampling were done from the last 40 ns of simulations. (**D**) SASA plots of RBD-ACE2 interface that calculated from all simulations of each system and represented in black, lime, orange and blue for models S and I–III respectively. The SASA plot of model D is shown in red.

The decreased fluctuations of O-glycosylated models II and III, does not necessarily prove stronger RBD-ACE2 interaction. However, calculating the distance between RBD-ACE2, RBD-H1, RBD-H2 and all the interacting pairs at the interface could provide hints on this mechanism. Distribution of the RBD-ACE2 distance values shows a similar pattern (peaks at 49 Å) in models III and S simulations (Fig. 9A). While, in models II, I and D simulations, RBD and ACE2 are by 1, 1.5 and 2Å more distant respectively (Fig. 9A). The distance fluctuation between RBD and ACE2 in model D is more altering. In addition to the ACE2-RBD, distances between RBD, H1, and H2 of ACE2 were also measured (Fig. 9B,C). Plots show that O-glycosylation always leads to a decrease between the RBD and the two helices (Fig. 9B,C). This decrease is more significant in H1-RBD distance for all O-glycosylated models compare to Model S (Fig. 9B). While, H2-RBD distance distribution also presents a decrease in the O-glycosylated models. Model D shows the largest distance values in both H1-RBD and H2-RBD plots(Fig. 9B,C). These observations suggest that RBD-ACE2 binding should be stronger at the direct interface upon O-glycosylation of S494 with elongated oligosaccharides. The SASA plots for the RBD-ACE2 interface show that model I is the most exposed in the binding interface(Figs. 9D and 9E )with an increase of about 1000 Å$^E2$ compared to other models. This is supportive of the distance distribution and RMSD/RMSF plots that showed a destabilized interaction in model I due to the small two-units glycan that increased the flexibility in the interface leading to form weak interactions with the neighbouring residues in model I.

**Figure 10:**
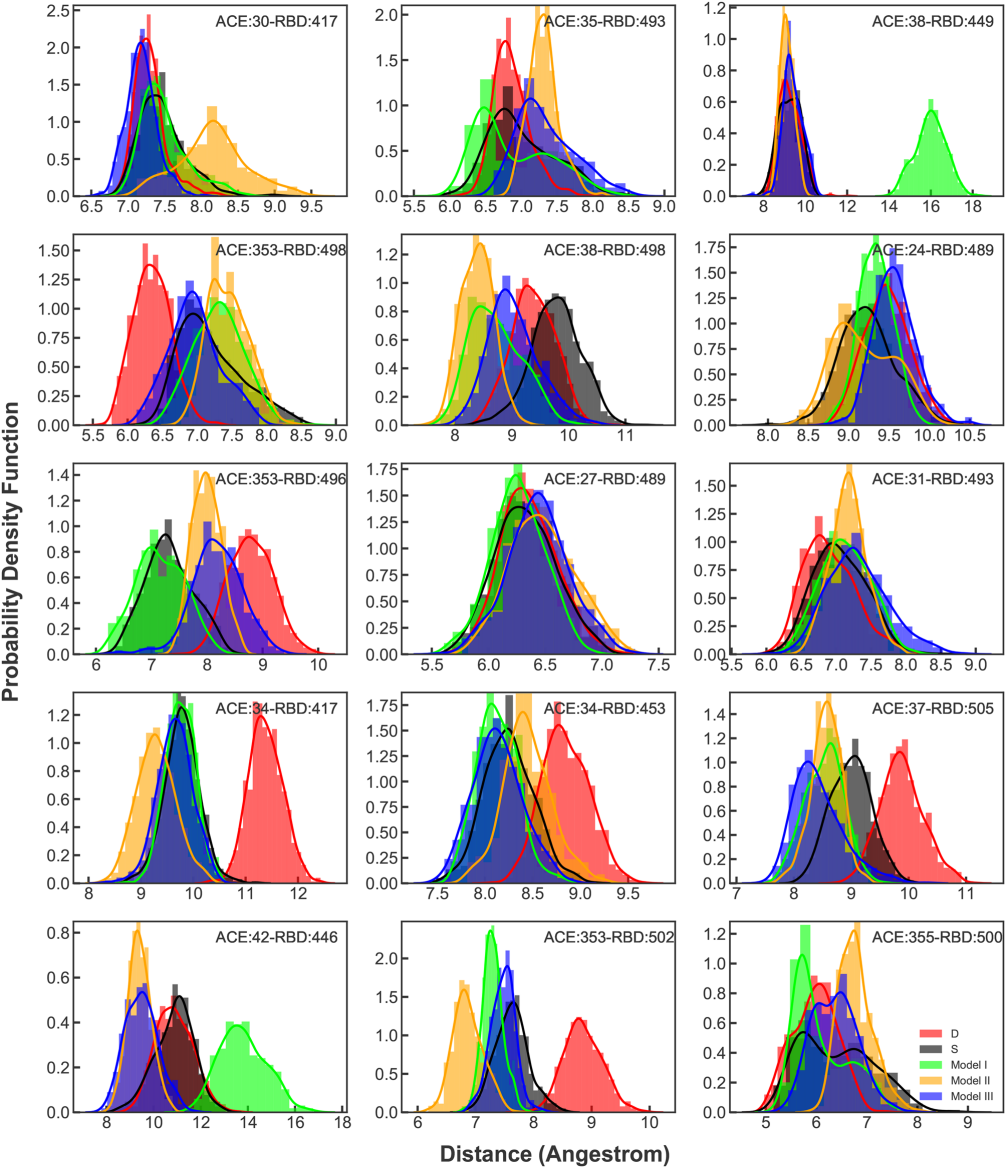
PDF of RBD-ACE2 interface interacting pairs averaged distance distribution with a histogram and maximum likelihood gaussian distribution fit: calculated from all replicate of each system and are represented in black, lime, orange and blue for models S and I–III respectively. PDFs of model D is shown in red.

The distance distribution plots of all the interacting pairs of residues on the RBD-ACE2 interface were also calculated (Fig. 10). The overall trend of the plots is very similar in models S, III and II. Showing that the main interactions in the interface are either not altered or strengthened by the O-glycans. This is due to the polar contacts between the glycans and the neighbouring residues that lead to stronger RBD-ACE2 interaction(Fig. 8). The most fluctuating distance distributions occur in model D. Where in 7 out of 15 interacting pairs (ACE38-RBD498, ACE353-RBD496, ACE34-RBD417, ACE34-RBD453, ACE37-RBD505, ACE42-RBD446, ACE353-RBD502), there is a noticeable increase in the peak location compared to other systems (Fig. 10). In most of the interacting pairs, the O-glycosylated models with elongated oligosaccharides show the smallest peak values (Fig. 10). In two of the interacting pairs (ACE353-RBD498, ACE31-RBD493 and ACE355-RBD500) model D shows a decrease in the distribution peaks (Fig. 10). The noticeable increases and decreases in model D plots for different interacting pairs show the system’s flexibility and weakened RBD-ACE2 interactions. Among the O-glycosylated models, I is the only system that present increased peak values for ACE38-RBD449 and ACE42-RBD446 interacting pairs. Model I was shown to be the most flexible O-glycosylated model (Fig. 3). Due to the flexibility that is added to the system by the short two-units glycan that is unable to form contacts with neighbouring residues in the interface in a manner that occurs for models II and III simulations (Fig. 8).

**Figure 11:**
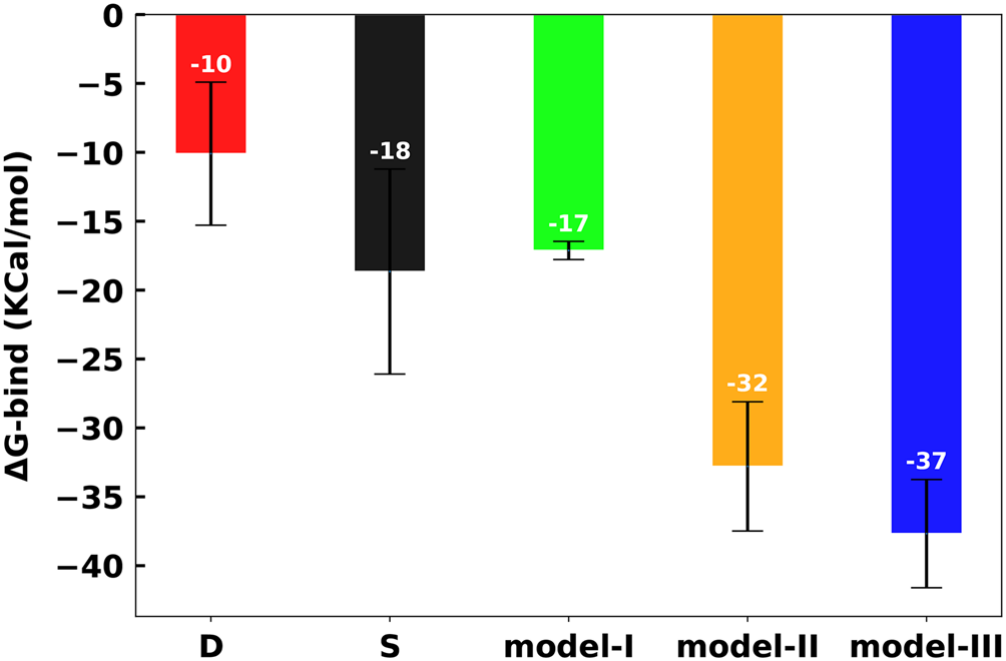
Relative binding free energy components for RBD-ACE2 complex. Overall binding free energy of models D, S, I–III are shown in red, black, lime, orange and blue.

The binding free energy between the RBD and ACE2 also shows that the interaction is most favorable upon O-glycosylation in models II and III with longer attached glycans (Fig. 11). The binding free energies between RBD-ACE2, calculated with the MMPBSA method, show a monotonic decrease in $\Delta$G by increasing the size of O-glycans (Fig. 11) and staring from − 17±0.66 kcal/mol for model I to − 32±4.69 kcal/mol and − 37.00±3.92 kcal/mol for models II and III respectively (Fig.  11). Control models S and D present less favourable binding energies. Model D shows the smallest $\Delta$G value − 10.00±5.193 kcal/mol among all systems. This is due to the dramatic destability of the complex upon S494D substitution that shows the appearance of S494 in the RBD of SARS-COV-2 promots the virion-ACE2 interaction. Interestingly, in model I with the core O-glycan the binding free energy is less favorable (-17±0.66 kcal/mol) compared to model S (-18±7.439 kcal/mol).

This is due to the increased flexibility at the ACE2-RBD binding interface and the decrease of polar interactions made by the core glycan due to its small length. RMSD and RMSF plots of the RBD and overall ACE2-RBD (Figs. 3, 4 and 6) are supportive of this observations and show an increase in the flexibility of the complex for model I. Chosen value of solute dielectric constant has shown to have a dramatic effect on the prediction of binding free energy, while using the MMPBSA method ^43–46^ (see method section of Supporting information). Regardless of the given value to the dielectric constant, the decrease of binding free energy in the O-glycosylated models II and III with longer glycans is always persistent and statistically significant (Fig. 11). The increase of the binding free energy in the glycosylated protein-ligand complexes are also reported in other studies^39,47^.

The stabilized dynamics and more favorable binding energy between ACE2 and RBD with elongated O-glycans attached, could increase the uptake of SARS-CoV-2 and the possibility of its high evasion. However, performing further simulations in parallel with experiments to test the effect of glycosylation on each residue in the binding interface could be a useful validation of the observations reported here.

## Conclusions

S494 that only occurs in SARS-CoV-2 and not in SARS-CoV is located in direct RBD-ACE2 binding interface has the potential to be decorated by O-glycans. Previous experimental and computational studies emphasized on the role of O-glycosylation in SARS-CoV-2 high infectivity. The results of atomistic molecular dynamics simulations of SARS-CoV-2 RBD in complex with ACE2 suggest that the O-glycosylation of S494 leads to the stronger interaction between RBD-ACE2 which can increase the virus infectivity. Three models of core and elongated O-glycans attached to RBD were tested and the results were compared with the unglcosylated S494 and S494D systems here. We observed that attachment of the elongated O-glycans lead to less flexible ACE2-RBD dynamics and decreased distances between RBD and two major H1, H2 helices of ACE2 that maintains the interacting pairs of amino acids in the direct binding interface. Relative binding free energy of RBD-ACE2 is also more favorable in the O-glycosylated models with longer glycans. The increase of RBD binding affinity to ACE2 depends on the size of attached O-glycan. By increasing the size of O-glycan, the RBD-ACE2 binding affinity will increase. These observations were confirmed by atomistic simulations of the unglycosylated S494 and the substitution of S494 to D that occurs in SARS-COV. Both of these system are more flexible with weakened interactions at the binding interface and less favourable binding affinity to ACE2.

The findings of this study add insightful information to the current status of SARS-CoV-2-ACE2 glycosylation and its role in the virus’s high evasion rate. This hypothesis is a suitable target for experimental validation, and if proven to be critical, must be considered in further therapeutic designs.

## Supplementary Information

Supplementary Information.

## References

[1] Andersen KG, Rambaut A, Lipkin WI, Holmes EC, Garry RF (2020). The proximal origin of SARS-CoV-2. Nat. Med..

[2] Wrapp D, Wang N, Corbett KS, Goldsmith JA, Hsieh C-L, Abiona O, Graham BS, McLellan JS (2020). Cryo-EM structure of the 2019-nCoV spike in the prefusion conformation. Science.

[3] Lan, J. *et al*. Crystal structure of the 2019-nCoV spike receptor-binding domain bound with the ACE2 receptor. bioRxiv (2020).

[4] Brielle, E. S., Schneidman-Duhovny, D. & Linial, M. The SARS-CoV-2 exerts a distinctive strategy for interacting with the ACE2 human receptor. bioRxiv (2020). 10.3390/v12050497 PMC7291053 32365751

[5] Kumar N, Sood D, Chandra R (2020). Vaccine formulation and optimization for human herpes virus-5 through an immunoinformatics framework. ACS Pharmacol. Transl. Sci..

[6] Kumar N, Sood D, van der Spek PJ, Sharma HS, Chandra R (2020). Molecular binding mechanism and pharmacology comparative analysis of noscapine for repurposing against SARS-CoV-2 protease. J. Proteome Res..

[7] Wang C, Li W, Drabek D, Okba NM, van Haperen R, Osterhaus AD, van Kuppeveld FJ, Haagmans BL, Grosveld F, Bosch B-J (2020). A human monoclonal antibody blocking SARS-CoV-2 infection. Nat. Commun..

[8] Catalan-Dibene, J. Human antibodies can neutralize SARS-CoV-2. *Nat Rev Immunol.**20*, 350 (2020). 10.1038/s41577-020-0313-6 (2020). 10.1038/s41577-020-0313-6 PMC7186926 32286538

[9] Yan R, Zhang Y, Li Y, Xia L, Guo Y, Zhou Q (2020). Structural basis for the recognition of SARS-CoV-2 by full-length human ACE2. Science.

[10] Amin, M., Sorour, M. K. & Kasry, A. Comparing the binding interactions in the receptor binding domains of SARS-CoV-2 and SARS-CoV. *J. Phys. Chem. Lett.* **11**(12), 4897–4900. 10.1021/acs.jpclett.0c01064 (2020). 10.1021/acs.jpclett.0c01064 32478523

[11] Han Y, Král P (2020). Computational design of ACE2-based peptide inhibitors of SARS-CoV-2. ACS Nano.

[12] He, J., Tao, H., Yan, Y., Huang, S.-Y. & Xiao, Y. Molecular mechanism of evolution and human infection with SARS-CoV- 2. *Viruses* **12**(4), 428. 10.3390/v12040428 (2020). 10.3390/v12040428 PMC7232534 32290077

[13] Tan J, Verschueren KH, Anand K, Shen J, Yang M, Xu Y, Rao Z, Bigalke J, Heisen B, Mesters JR, Chen K, Shen X, Jiang H, Hilgenfeld R (2005). pH-dependent conformational flexibility of the SARS-CoV main proteinase (Mpro) dimer: Molecular dynamics simulations and multiple x-ray structure analyses. J. Mol. Biol..

[14] Rath, S. L. & Kumar, K. Investigation of the effect of temperature on the structure of SARS-Cov-2 spike protein by molecular dynamics simulations. *Front Mol Biosci.* **7**, 583523. 10.3389/fmolb.2020.583523 (2020). 10.3389/fmolb.2020.583523 PMC7596554 33195427

[15] Mariño K, Bones J, Kattla JJ, Rudd PM (2010). A systematic approach to protein glycosylation analysis: A path through the maze. Nat. Chem. Biol..

[16] Shajahan, A., Supekar, N. T., Gleinich, A. S. & Azadi, P. Deducing the N-and O-glycosylation profile of the spike protein of novel coronavirus SARS-CoV-2. *Glycobiology*. **30**(12), 981–988. 10.1093/glycob/cwaa042 (2020). 10.1093/glycob/cwaa042 PMC7239183 32363391

[17] Zhao, P. *et al*. Virus-receptor interactions of glycosylated SARS-CoV-2 spike and human ACE2 receptor. *Cell host Microbe* **28**, 586–601 (2020). 10.1016/j.chom.2020.08.004 PMC7443692 32841605

[18] Fung TS, Liu DX (2018). Post-translational modifications of coronavirus proteins: Roles and function. Future Virol..

[19] Parsons LM, Bouwman KM, Azurmendi H, De Vries RP, Cipollo JF, Verheije MH (2019). Glycosylation of the viral attachment protein of avian coronavirus is essential for host cell and receptor binding. J. Biol. Chem..

[20] Uslupehlivan, M. & Şener, E. Glycoinformatics approach for identifying target positions to inhibit initial binding of SARS-CoV-2 S1 protein to the host cell. bioRxiv (2020).

[21] Watanabe, Y., Bowden, T. A., Wilson, I. A. & Crispin, M. Exploitation of glycosylation in enveloped virus pathobiology. Biochimica et Biophysica Acta (BBA)-General Subjects. *Biochim Biophys Acta Gen Subj*. **1863**(10), 1480–1497. 10.1016/j.bbagen.2019.05.012 (2019). 10.1016/j.bbagen.2019.05.012 PMC6686077 31121217

[22] Hounsell EF, Davies MJ, Renouf DV (1996). O-linked protein glycosylation structure and function. Glycoconjugate J..

[23] Singh, K. & Tripathi, R. P. An overview on glyco-macrocycles: Potential new lead and their future in medicinal chemistry. *Curr. Med. Chem.* **27**(20), 3386–3410. 10.2174/0929867326666190227232721 (2020). 10.2174/0929867326666190227232721 30827227

[24] Ridley C, Thornton DJ (2018). Mucins: The frontline defence of the lung. Biochem. Soc. Trans..

[25] Lamblin G, Lhermitte M, Klein A, Houdret N, Scharfman A, Ramphal R, Roussel P (1991). The carbohydrate diversity of human respiratory mucins: A protection of the underlying mucosa. Am. Rev. Respir. Dis..

[26] Cristelo, C., Azevedo, C., Marques, J. M., Nunes, R. & Sarmento, B. SARS-CoV-2 and diabetes: New challenges for the disease. *Diabetes Res. Clin. Pract.* **108228**, (2020). 10.1016/j.diabres.2020.108228 PMC7242186 32446801

[27] Kirschner KN, Yongye AB, Tschampel SM, González-Outeiriño J, Daniels CR, Foley BL, Woods RJ (2008). GLYCAM06: A generalizable biomolecular force field. Carbohydrates. J. Comput. Chem..

[28] Jorgensen WL, Chandrasekhar J, Madura JD, Impey RW, Klein ML (1983). Comparison of simple potential functions for simulating liquid water. J. Chem. Phys..

[29] Klauda JB, Venable RM, Freites JA, O’Connor JW, Tobias DJ, Mondragon-Ramirez C, Vorobyov I, MacKerell AD, Pastor RW (2010). Update of the CHARMM all-atom additive force field for lipids: Validation on six lipid types. J. Phys. Chem. B.

[30] Huang J, Rauscher S, Nawrocki G, Ran T, Feig M, de Groot BL, Grubmüller H, MacKerell AD (2017). CHARMM36m: An improved force field for folded and intrinsically disordered proteins. Nat. methods.

[31] Phillips JC, Braun R, Wang W, Gumbart J, Tajkhorshid E, Villa E, Chipot C, Skeel RD, Kale L, Schulten K (2005). Scalable molecular dynamics with NAMD. J. Comput. Chem..

[32] Darden T, York D, Pedersen L (1993). Particle mesh Ewald: An N log (N) method for Ewald sums in large systems. J. Chem. Phys..

[33] Liu H, Hou T (2016). CaFE: A tool for binding affinity prediction using end-point free energy methods. Bioinformatics.

[34] Baker NA, Sept D, Joseph S, Holst MJ, McCammon JA (2001). Electrostatics of nanosystems: Application to microtubules and the ribosome. Proc. Natl. Acad. Sci..

[35] Humphrey W, Dalke A, Schulten K (1996). VMD—Visual molecular dynamics. J. Mol. Graph..

[36] Hunter JD (2007). Matplotlib: A 2D graphics environment. Comput. Sci. Eng..

[37] Van Rossum, G. & Drake, F. L. *Python 3 Reference Manual* (CreateSpace, 2009).

[38] Shental-Bechor D, Levy Y (2008). Effect of glycosylation on protein folding: A close look at thermodynamic stabilization. Proc. Natl. Acad. Sci..

[39] Azimzadeh Irani, M., Kannan, S. & Verma, C. Role of N-glycosylation in EGFR ectodomain ligand binding. *Proteins*, **85**, 1529–1549. 10.1002/prot.25314 (2017). 10.1002/prot.25314 28486782

[40] Azimzadeh Irani, M., & Ejtehadi, M. R. Glycan-mediated functional assembly of IL-1RI: Structural insights into completion of the current description for immune response. *J. Biomol. Struct. Dyn.*10.1080/07391102.2020.1841027 (2020). 10.1080/07391102.2020.1841027 33124956

[41] Ali A, Vijayan R (2020). Dynamics of the ACE2-SARS-CoV-2/SARS-CoV spike protein interface reveal unique mechanisms. Sci. Rep..

[42] Lan, J. *et al*. Structure of the SARS-CoV-2 spike receptor-binding domain bound to the ACE2 receptor. *Nature* **581**, 215–220 (2020). 10.1038/s41586-020-2180-5 32225176

[43] Li Y, Cong Y, Feng G, Zhong S, Zhang JZ, Sun H, Duan L (2018). The impact of interior dielectric constant and entropic change on HIV-1 complex binding free energy prediction. Struct. Dyn..

[44] Hou, T., Wang, J., Li, Y., Wang, W. Assessing the performance of the MM/PBSA and MM/GBSA methods. 1. The accuracy of binding free energy calculations based on molecular dynamics simulations. *J. Chem. Inf. Model.* **51**, 69–82 (2011). 10.1021/ci100275a PMC3029230 21117705

[45] Srivastava HK, Sastry GN (2012). Molecular dynamics investigation on a series of HIV protease inhibitors: Assessing the performance of MM-PBSA and MM-GBSA approaches. J. Chem. Inf. Model..

[46] Kumar, N. *et al.* Antitussive noscapine and antiviral drug conjugates as arsenal against COVID-19: A comprehensive chemoinformatics analysis. *J. Biomol. Struct. Dyn.* 1–16 (2020) (**PMID: 32815796**). 10.1080/07391102.2020.1808072 PMC7484584 32815796

[47] Azimzadeh Irani M, Ejtehadi MR (2019). GAG positioning on IL-1RI; A mechanism regulated by dual effect of glycosylation. Glycobiology.

