# Supplementary material for: S494 O-glycosylation site on the SARS-CoV-2 RBD affects the virus affinity to ACE2 and its infectivity; a molecular dynamics study
Source: Sci Rep. 2021 Jul 26;11:15162. doi: 10.1038/s41598-021-94602-w (PMC8313699; doi:10.1038/s41598-021-94602-w)
Supplement: Supplementary file 1 — Supplementary Information. [file 41598_2021_94602_MOESM1_ESM.pdf]

Supplementary Information: S494 O-glycosylation site on the SARS-CoV-2 RBD Affects the Virus Affinity to ACE2 and its Infectivity; A Molecular Dynamics Study

Shadi Rahnama,† Maryam Azimzadeh Irani,*,‡ Mehriar Amininasab,¶ and Mohammad Reza Ejtehadi*,§

†*Institute for Nanoscience and Nanotechnology, Sharif University of Technology, Tehran 14588, Iran.*

‡*Faculty of Life Sciences and Biotechnology, Shahid Beheshti University, Tehran, Iran.*

¶*Department of Cell and Molecular Biology, School of Biology, College of Science, University of Tehran, Tehran, Iran.*

§*Department of Physics, Sharif University of Technology, Tehran 14588, Iran.*

E-mail: m_azimzadeh@sbu.ac.ir; ejtehadi@sharif.edu

Phone: +98 (0)21 29901; +98 (0)21 6616 4525. Fax: +98 (0)21 22431919; +98 (0)21 6602 2711

Supplementary Method

For MMPBSA binding energy calculation ligand-receptor complex is first calculated in the gas state and then in solvated state. The overall relative binding free energy is the sum of those two ΔGs (Eq.1). Each ΔG can be decomposed into the contribution of different

interactions and expressed in:[1]

$$\Delta G_{bind} = \Delta G_{gas} + \Delta G_{sol} \tag{1}$$

$$\Delta G_{gas} = \langle \Delta G_{mm} \rangle - T\Delta S \tag{2}$$

$$\Delta G_{sol} = \Delta G_{PB} + \Delta G_{SA} \tag{3}$$

Where $\langle \Delta G_{mm} \rangle$, $-T\Delta S$, ΔG_{PB} and ΔG_{SA} are the ensemble-averaged protein-ligand interactions, entropy contributions, electrostatic, and nonpolar solvation energies, respectively. For calculating the entropic part of the binding energy, Interaction Entropy (IE) method[2] was used:

$$-T\Delta S = KT \ln\langle e^{\Delta E_{pl}^{int}} \rangle \tag{4}$$

where ΔE_{pl}^{int} represents the fluctuation of protein-ligand interaction energy around the average energy and calculated as follow:[2]

$$\Delta E_{pl}^{int} = E_{pl}^{int} - \langle \Delta E_{pl}^{int} \rangle \tag{5}$$

where $\langle \Delta E_{pl}^{int} \rangle$ represents the average of protein-ligand interaction energy.

The 1.2 value for the dielectric constant in the binding free energy calculation was selected because it produced the closest values to the experimental RBD-ACE2 binding energy[3] among all the other commonly used tested values. Also it has shown to be a reasonable value for such calculations according to the proposed fitting curve by Tingjun Hou et al.[4]

Supplementary Tables

Table S1: N-Glycan composition for ACE2 and RBD

	Site	Type	Structure	Sequence
Ace2	N53	High mannose (core)	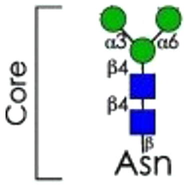	aDMan(1→6)[aDMan(1→3)]bDMan(1→4) bDGlcNAc(1→4)bDGlcNAc(1→)PROA
Ace2	N90	High mannose (core)		
Ace2	N103	High mannose (core)		
Ace2	N322	High mannose (core)		
Ace2	N432	High mannose (core)		
Ace2	N546	High mannose (core)		
RBD	N343	High mannose (core)		

Table S2: O-Glycan composition for RBD

Site	Model	Structure	Sequence
S494	I	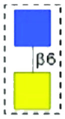	bDGlcNAc(1→6)bDGalNAc(1→)PROA-494
S494	II	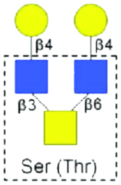	bDGal(1→4)bDGlcNAc(1→6)[bDGal(1→1) bDGlcNAc(1→3)]bDGalNAc(1→)PROA-494
S494	III	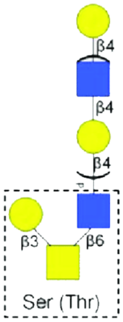	bDGal(1→4)bDGlcNAc(1→4)bDGal(1→4) bDGlcNAc(1→6)[bDGal(1→3)]bDGalNAc(1→)PRO A-494

Supplementary Figures

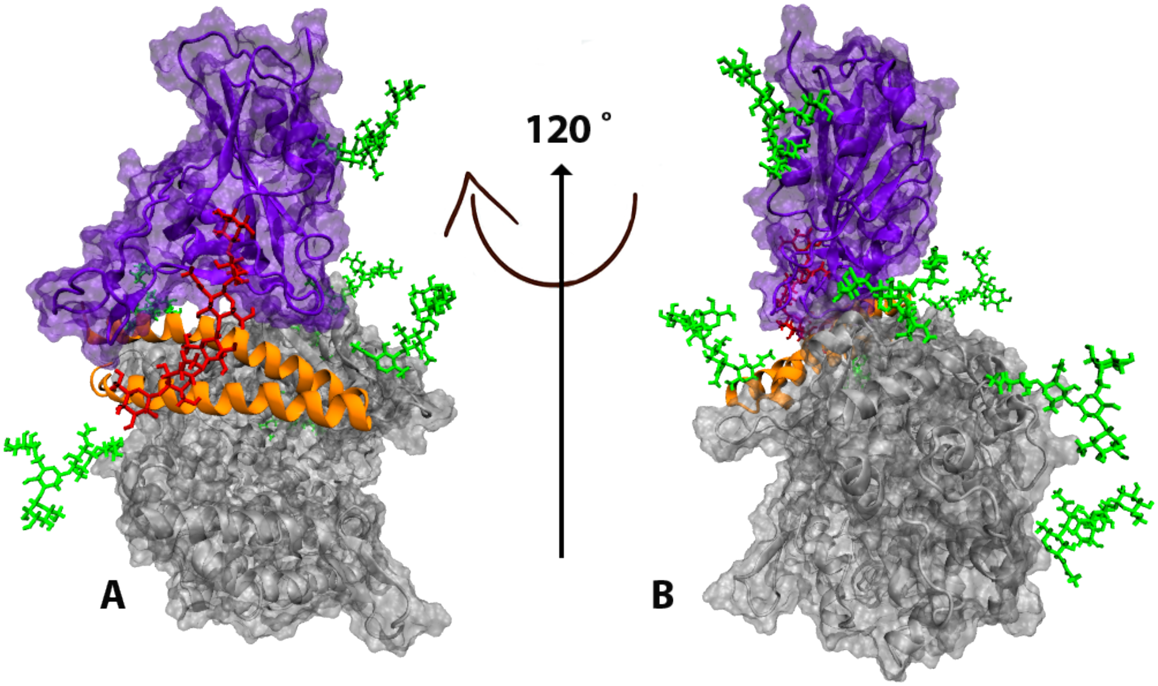

Figure S1: Visualization of the complex with full presentation of the N-glycans. 120° Z-axis rotation of model in A was presented in B. The extracellular domain of human ACE2 and RBD of SARS-COV-2 S protein is shown with gray and purple, respectively. The N-glycan are shown with green sticks. Red stick is model III o-glycan.

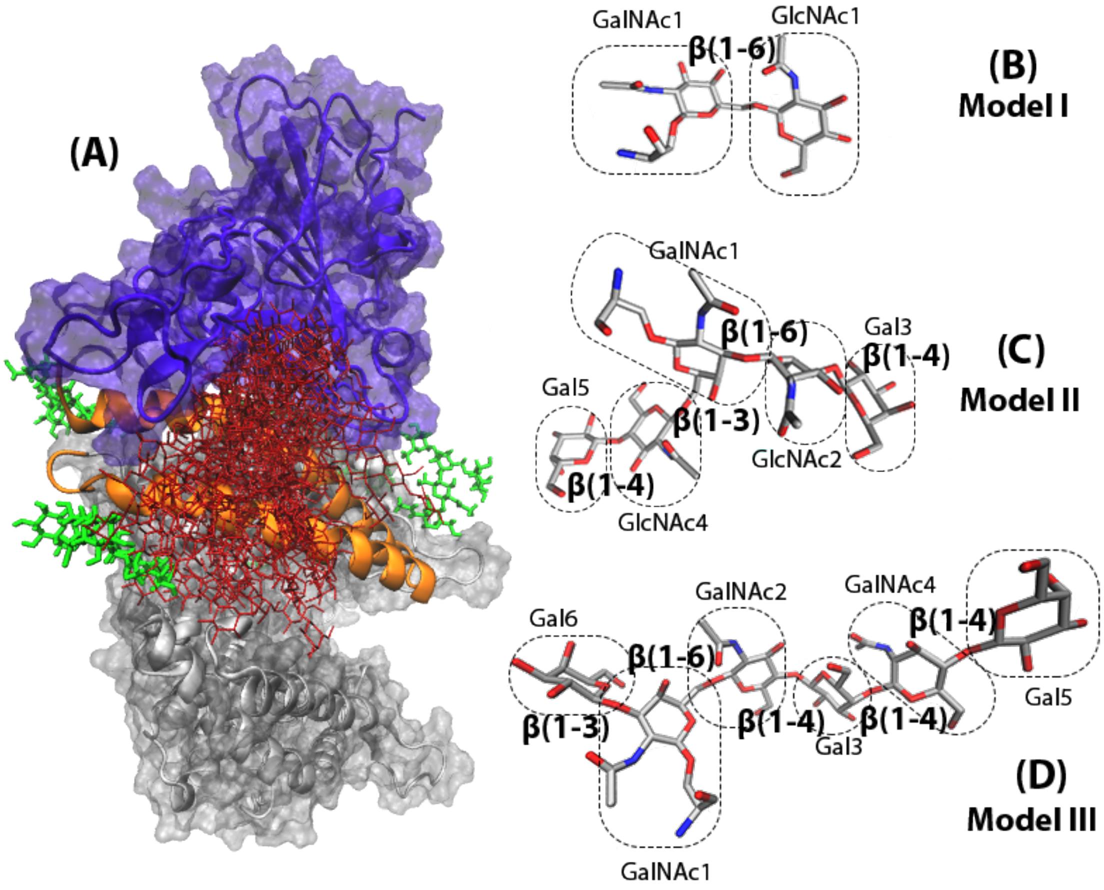

Figure S2: (A) The overlaid presentation of O-glycosylated RBD-ACE2 complex is shown with red sticks. The extracellular domain of human ACE2 and RBD of SARS-CoV-2 S protein is shown with gray and purple, respectively. O-glycan models I, II and III and their internal linkage are shown with label B, C and D respectively.

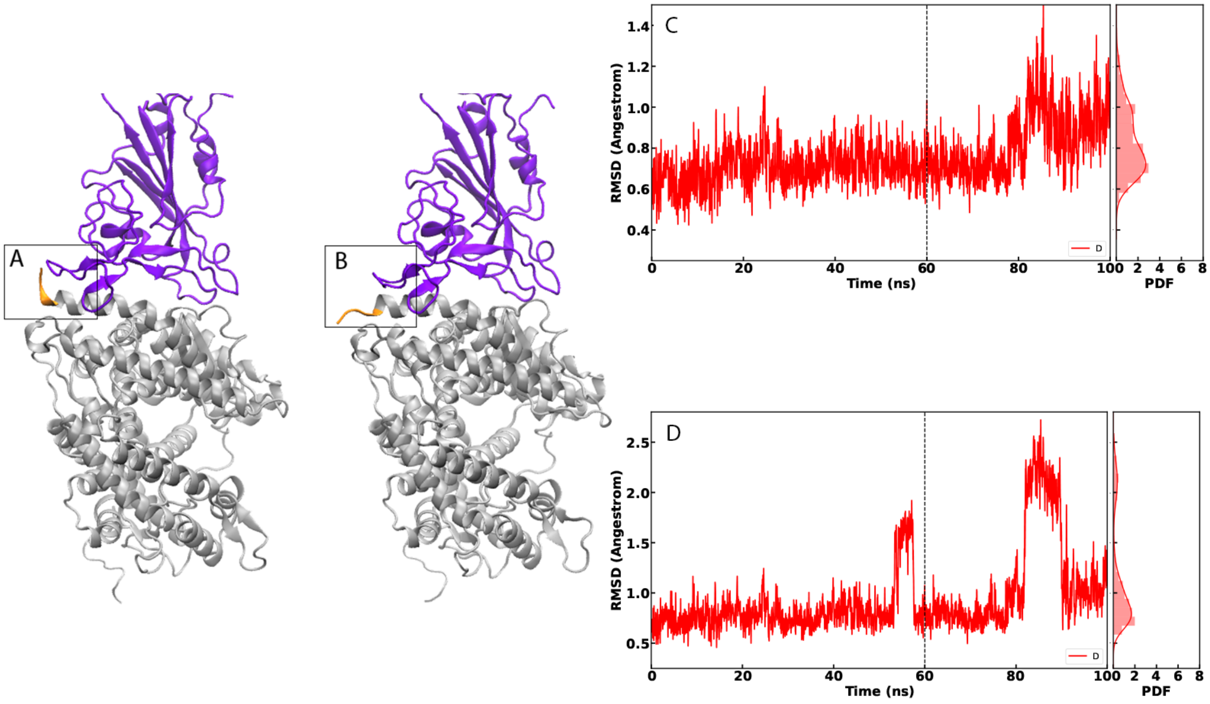

Figure S3: The conformational change in residue 19-22 from state A to B, in one of replicas of model D, resulted in visible jumps in RMSD plots (D). Without considering these dangling residue, these jumps are diaapeared (C).

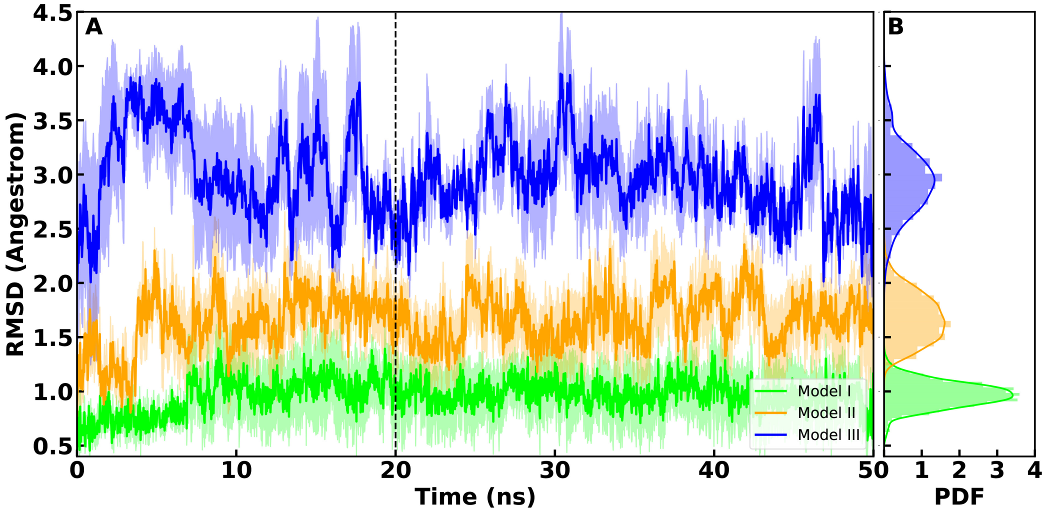

Figure S4: (A) Average RMSD plots for three models of O-glycans calculated from all replicates of each system and are shown for model I (lime), model II (orange) and model III (blue). Light shades around each plot presents standard error for each calculation. (B) Probability Density Function (PDF) of RMSD sampled over the last 30 ns (dashed line) of the simulations are shown in histograms.

References

(1) Genheden, S.; Ryde, U. The MM/PBSA and MM/GBSA methods to estimate ligand-binding affinities. *Expert opinion on drug discovery* **2015**, *10*, 449–461.

(2) Duan, L.; Liu, X.; Zhang, J. Z. Interaction entropy: a new paradigm for highly efficient and reliable computation of protein–ligand binding free energy. *Journal of the American Chemical Society* **2016**, *138*, 5722–5728.

(3) Wrapp, D.; Wang, N.; Corbett, K. S.; Goldsmith, J. A.; Hsieh, C.-L.; Abiona, O.; Graham, B. S.; McLellan, J. S. Cryo-EM structure of the 2019-nCoV spike in the prefusion conformation. *Science* **2020**, *367*, 1260–1263.

(4) Hou, T.; Wang, J.; Li, Y.; Wang, W. Assessing the performance of the MM/PBSA and MM/GBSA methods. 1. The accuracy of binding free energy calculations based on

molecular dynamics simulations. *Journal of chemical information and modeling* **2011**, *51*, 69–82.
